# Mold, Mycotoxins and a Dysregulated Immune System: A Combination of Concern?

**DOI:** 10.3390/ijms222212269

**Published:** 2021-11-12

**Authors:** Stephanie Kraft, Lisa Buchenauer, Tobias Polte

**Affiliations:** 1 Rudolf-Boehm-Institute for Pharmacology and Toxicology, Leipzig University, 04107 Leipzig, Germany; 2 Department for Environmental Immunology, Helmholtz Centre for Environmental Research—UFZ, 04318 Leipzig, Germany; lisa.buchenauer@ufz.de; 3 Department of Dermatology, Venerology and Allergology, Leipzig University Medical Center, Leipzig University, 04103 Leipzig, Germany

**Keywords:** molds, mycotoxins, dysfunctional immune system, immune system disorders, immune response

## Abstract

Fungi represent one of the most diverse and abundant eukaryotes on earth. The interplay between mold exposure and the host immune system is still not fully elucidated. Literature research focusing on up-to-date publications is providing a heterogenous picture of evidence and opinions regarding the role of mold and mycotoxins in the development of immune diseases. While the induction of allergic immune responses by molds is generally acknowledged, other direct health effects like the toxic mold syndrome are controversially discussed. However, recent observations indicate a particular importance of mold/mycotoxin exposure in individuals with pre-existing dysregulation of the immune system, due to exacerbation of underlying pathophysiology including allergic and non-allergic chronic inflammatory diseases, autoimmune disorders, and even human immunodeficiency virus (HIV) disease progression. In this review, we focus on the impact of mycotoxins regarding their impact on disease progression in pre-existing immune dysregulation. This is complemented by experimental in vivo and in vitro findings to present cellular and molecular modes of action. Furthermore, we discuss hypothetical mechanisms of action, where evidence is missing since much remains to be discovered.

## 1. Introduction

Humans are exposed to multiple fungi. Many fungi live in harmony with humans, rarely causing diseases. Possible health effects associated with fungi generally fall into the category infections (mycosis), allergic reactions, or toxic effects [1]. Fungi causing mycosis include dermatophytes, yeast, and mold. Fungal infection requires exposure to spores via inhalation, ingestion of contaminated food, or skin contact [2,3]. Although fungal infection can occur in healthy individuals, especially individuals with a weakened immune system are of risk (reviewed by Kohler et al. [4]).

Molds are fungi, which grow in form of multicellular filaments called hyphae and are found in distinct environments: air, soil, plants, animals, and human hosts [5,6]. It is present indoors and outdoors and grows in places with a lot of moisture [7]. Mold exposure is reported for contaminated plant-based foods, carried-over mycotoxin metabolites to meat and dairy products, air and dust [8], predominantly in countries with lacking implementation of adequate food safety policies [8]. Mycotoxins are diverse toxic secondary metabolites that are naturally produced by a wide range of molds [9]. Notably one mold species can produce several mycotoxins, and vice versa, different mold genie may produce the same mycotoxin [9,10].

Significant exposure to mycotoxins has also been found in developed countries [11], even in highly controlled infant food products [12]. Moreover, mycotoxins were detected in children’s plasma [13]. Additionally, occupational exposure to carcinogenic aflatoxins (AF) in agricultural-related workspaces worldwide are a well-known health concern [14]. To date, several hundred mycotoxins have been identified. Various high-performance liquid chromatography techniques like liquid chromatography/mass spectrometry (LC/MS) are used for identification and detection of mycotoxins. Particularly, LC coupled to tandem mass spectrometry (LC/MS/MS) has become important for mycotoxin analysis [15]. Additionally, molecular polymerase chain reaction(PCR) approaches have been developed [15]. Moreover, enzyme-linked immunosorbent assays (ELISA) are on the market to detect mycotoxins in different matrices including urine samples.

In the past decade, mycotoxins have attracted considerable attention due to their potential of strong toxicity. Harmful effects caused by mycotoxins include acute poisoning after consumption of high dosages, which may even result in death [16]. AF, as the most prominent example, can cause irreversible liver damage [16]. Furthermore, adverse effects on many internal organs have been reported [16]. The respiratory system is the first point of contact during inhalation of mold spores and mycotoxins. Mycotoxins like AF have been shown in vivo to slow down basal and stimulate respiratory ciliary beat frequency, potentially increasing pathogenesis and infection by impairment of clearance [17]. Furthermore, damaging effects of mycotoxins on the gastrointestinal tract due to ingestion of contaminated food have been described, commonly manifested as inflammation, necrotic changes, damage to intestinal barrier function, impairment of secretory activity and alterations in enterocyte metabolism (reviewed by Gonkowski et al. [18]). Initial in vivo studies also indicate an influence on the enteric nervous system (ENS) via gastrointestinal tract contact [18]. Previous review articles summarized that exposure to molds, mycotoxins and water damaged buildings might also be associated with neurological and neuropsychiatric symptoms including pain, movement difficulty, delirium dementia and disorders of balance and coordination disorders [19]. Currently, the contribution of fungal opportunistic infections secreting mycotoxins into the nervous system and its neurotoxic effects in amyotrophic lateral sclerosis (ALS) is under investigation [20]. The combination of inflammation from the chronic infection and production of neurotoxins might be involved in systemic neuronal degeneration [20]. Even a connection between mycotoxins and Parkinson’s disease has been observed [21]. On the other hand, two recent reviews [22,23] critically discussed a health issue describes as “toxic mold syndrome”. These review articles conclude that there is currently no evidence for a causative relationship between the occurrence of mold and the described symptoms [22,23].

The immune system plays a key role in host–pathogen interaction and is essential for protecting living organisms against pathogens like molds and mycotoxins. Intact barrier functions, innate, and adaptive immune responses are essential for effective and successful elimination of infectious pathogens and toxic metabolites. These mechanisms might be disturbed in response to fungal cell wall antigens, or immunomodulatory and immunotoxic actions of mycotoxins, and therefore are critical for the development or exacerbation of immune system-related diseases.

The present review aims to indicate that, next to direct effects of mold and mycotoxin exposure in immunocompetent individuals, exposure in the presence of pre-existing immune dysregulation is of particular concern. First, we briefly describe how mycotoxins directly exert immunomodulatory effects. Next, we illustrate how mycotoxins might trigger the onset or exacerbation of chronic inflammatory diseases, autoimmune disorders, and progression of HIV disease [24,25,26,27,28,29]. Furthermore, cellular, and molecular key mechanisms of these exacerbating effects in the progression of the underlying diseases are discussed.

## 2. Mold-Induced Immunological Effects

### 2.1. Toxic Mold Syndrome

The toxic mold syndrome is described as a symptom complex including various vague characteristics like cognitive impairment, emotional disturbance, respiratory complaints but also symptoms like muscle ache. It is thought to be a direct effect of indoor “black mold” and its mycotoxins [23]. Nevertheless, to date, there is a critical debate going on about this issue, since scientific evidence is lacking [22,23]. According to the critical voices, one would assume that the “toxic mold syndrome” is a nocebo effect to visible “black mold” and not a real subject of public health concern. However, molds and their mycotoxins have been shown to induce direct hypersensitive reactions and have immunomodulatory properties in immunocompetent individuals. Moreover, there is growing evidence that mycotoxins are of specific concern for individuals with a pre-existing impairment of the immune system.

### 2.2. Mold-Induced Hypersensitivity

Mold can cause different hypersensitivity reactions. Hypersensitivity reactions are exaggerated or inappropriate immunologic responses occurring in response to an allergen [30]. Allergens act as antigens resulting in a strong immune response. The four genera *Alternaria* spp., *Cladosporium* spp., *Aspergillus* spp. and *Penicillium* spp. belong to the phylum Ascomycota for which currently 88 allergens are described (see www.allergen.org; accessed on 30 August 2021). Allergens include enolase, heat shock proteins, cyclophilins, proteases, redoxins, and disulphide isomerases [31,32]. According to our current knowledge, there is no evidence that mycotoxins may function as allergens.

Type 1 allergies are characterized by antigen-specific IgE-antibodies, which are produced shortly after exposure to already sensitized mold antigens. Not all the mold produced IgE-binding antigens are equally important [33]. Although the exact prevalence is unknown, it is estimated to range from 3 to 10% [33]. Even if the prevalence of sensitization can only be estimated, there are multiple studies indicating, mold is considered an important allergen source for allergic asthma [34].

Recent studies indicate, that classical outdoor species like *A. alternata* and *A. fumigatus,* as well as various indoor mold species exert strong inflammatory and allergenic properties in asthmatics with mold sensitization [24]. Next to allergic asthma, fungal rhinosinusitis (AFRS) a subset of chronic rhinosinusitis with nasal polyps (CRSwNP) has also been classified as type 1 allergy with elevated levels of mold-specific IgE [35].

In addition to type 1 allergies, mold can also induce type 3 and 4 hypersensitive reactions, known as hypersensitivity pneumonitis (HP) [36]. These reactions are mediated by immune complexes and T helper 1 cells, respectively [37,38]. HP has been associated with airway exposure to high concentrations of mold spores, especially due to occupational conditions like farmer’s lung, or trombone player’s lung in wind musicians [25,26,36,39].

### 2.3. Immune Modulatory Potential of Mycotoxins

Mycotoxins occurring in food have been associated with long-term health effects like immune deficiency, which can result in an increased risk of infection susceptibility and cancer [16]. A recent review article addressing aflatoxin B1 (AFB1) summarized the acute and chronic effects including immunotoxicity of AF in humans and animals. The mechanisms of action underlying immunotoxicity of AF are still under investigation. AF can induce immunosuppressive but also immunostimulatory effects [40]. A recent in vivo study showed, that aflatoxin M1 (AFM1) suppressed innate and acquired immunity [41]. Patulin, a mycotoxin found in several fruits and their products such as juice or jams, has been shown to suppresses innate immune responses in vitro [42]. Furthermore, the mycotoxin ochratoxin A (OTA) mainly occurring in cereals, coffee, and red wine, has been reported to affect immunological response in piglets [43]. In humans, it is predominantly known for its acute nephrotoxic effect and a range of chronic disorders like upper urothelial carcinoma [44], but also immunomodulatory effects are under discussion [45].

Contamination with the *Fusarium* spp. mycotoxin deoxynivalenol (DON) in food is a public health concern according to WHO [16]. To date it is still unclear whether DON exposure influences certain diseases such as allergies.

## 3. Mold-Induced Exacerbation of Immune System Disorders

### 3.1. Mold Exposure and Increased Asthma Severity

Asthma is a very heterogeneous disease, characterized by airway inflammation and hyperreactivity. Asthma pathogenesis is complex and can be induced by allergens, non-allergens, and intrinsic factors [46]. Irrespectively from etiology, asthma can be categorized into different severity phenotypes with mild-to-moderate versus severe asthma. According to ERS/ATS 2014 guidelines, severe asthma is defined as difficult-to-treat asthma, accompanied by either lung impairment and/or risk of exacerbation [47,48].

Current knowledge about mold exposure and association with asthma was analyzed in a meta-analysis, including 148 studies [49]. Dampness or mold exposure was associated with increased asthma development and exacerbation in allergic and nonallergic individuals [49]. Mold exposure might modulate asthma severity in two aspects, (1) as sensitizer associated with increased severity of allergic asthma [50], and (2) as a pathogen related to severe asthma by a non-specific inflammatory mechanisms [50].

First, *A. fumigatus* has recently been shown to be of special concern as a sensitizer associated with increased allergic asthma severity (Table 1) [50].

In a case control study of Vincent et al., 2018 [50] 64 asthmatic subjects were classified as mold-sensitized asthmatic cases (positive skin prick test and/or CAP test to mold) or asthmatics without mold sensitization (negative for all skin prick tests and/or CAP test). In *A. fumigatus* sensitized asthmatics elevated total IgE, a higher degree of broncho-obstruction (FEV_1_/FVC) and a tendency of increased risk for severe asthma were detected in comparison to asthmatics without mold-sensitization [50]. Regarding the used surrogate markers in the presented human studies [50] (Table 1), total serum IgE-measurement was used as standard measurement to assess possible allergy. Notably, the clinical significance of an antigen does not lie in its IgE-binding capacity but in its capability to induce strong IgE-medicated and T-cell-mediated reactions [33]. These reactions should be assessed in vitro by measurement of basophil histamine release and in vivo skin and provocation test. Moreover, T-cell mediated responses are measured in vitro by T-cell proliferation assay and in vivo via atopy patch test [33]. Therefore, serum total IgE-measurement is a weak readout for the assessment of an ongoing allergic reaction. The FEV1/FVC ratio, also called Tiffeneau-Pinelli index, is a calculated ratio used in the diagnosis of obstructive and restrictive lung disease (e.g., asthma). The advantage lies in the broad application and comparison of this parameter between different studies. Moreover, determination of suspected mycotoxins and/or their metabolites in serum or urine samples would have increased the impact of the discussed studies (Table 1).

The findings of the human study by Vincent et al., 2018 [50] are in line with in vivo findings in a mouse model, showing *A. fumigatus* to be more pro-allergenic (Th2 allergic response) compared to other mold species [24]. Taken together, at least for *A. fumigatus* an association between exposure and severe allergic asthma phenotype due to increased sensitization does exist. However, to date there is no consensus on whether higher sensitization to allergens in general leads to an increased risk for the development of severe asthma [51]. According to Fitzpatrick et al., 2006 [52] severe asthma in children had a more profound IgE sensitization and more positive skin prick tests for different allergens (weed mix, *D. farinae*, and *D. pteryn*) than those of mild-to-moderate asthma phenotype children [52]. Of note, the two study groups did not differ in sensitization to animal dander, tree, or mold [52]. Mendell et al., 2011 [49] summarized in their meta-analyses, that there is strong evidence for dampness and mold to cause asthma exacerbation in children. On the contrary, severe asthma was accompanied by less sensitization in adults [52]. Zhang et al., 2018 [53] recently addressed the question, whether fungi including mold act as allergens when they exert their impact on allergic inflammation and came to the conclusion, that although allergens play an important role in the promotion of asthma, additional factors that act as immunomodulators probably contribute the exacerbation of asthma. Mycotoxins might be considered as one such factor. In vivo, it has been demonstrated that worsening of allergic asthma hallmarks was related to mycotoxin exposure in the absence of mold allergens [54]. Exposure of ovalbimun (OVA)-sensitized mice to gliotoxin (GTX) and patulin significantly increased airway inflammation, the number of eosinophils in bronchoalveolar lavage and the specific OVA-IgE levels compared to mycotoxin unexposed OVA-sensitized animals.

Next to the role of mold in severe allergic asthma, *Penicillium* spp. indoor exposure was also related to increased asthma severity in non-sensitized asthmatics in a small number of cases (Table 1) [50]. This effect could be explained by non-specific inflammatory mechanisms. However, due to small sample size verification is necessary [50].

In general, it is currently hypothesized, that the risk factor for increased asthma severity might not be due to sui generis of mold [50]. This is interesting, since one mold species can produce several mycotoxins, and vice versa different mold genie may produce the same mycotoxin. Overall, recent findings strengthen the evidence that mold exposure might be one of multiple risk factors regarding the development of severe asthma with exacerbation of asthmatic symptoms and impairment of lung function. Mold allergens are suspected to be allergic triggers [47]. However, mycotoxins might be considered of significant relevance in the exacerbation of asthma irrespective of its etiology [54].

### 3.2. Mycotoxin Exposure and Its Association with Autoimmune Disorders

The pathogenesis of autoimmune diseases is multifactorial with both genetic and environmental factors playing a role. In addition, epigenetic modifications can be triggered by environmental exposures to cause aberrant expression of genes and induce autoimmune diseases.

Multiple sclerosis (MS) is characterized by neuroinflammation and axonal demyelination of neurons in the central nervous system and spinal cord. A correlation between fungal infection and MS has been described for yeast *Candida* spp. [28]. Irrespective of MS diagnosis, neural protein autoantibodies were increased in several individuals, which were exposed to mold [55,56]. Similarly, a conducted cohort study of 8 females with known exposure to water-damaged or mold-contaminated buildings were tested positive for IgG neuronal antibodies against microtubule-associated protein-2, myelin basic protein, tau, glial fibrillary acidic protein, tubulin, and S-100B (Table 1) [57]. Nevertheless, these results must be considered with caution, since mycotoxin measurements in patients serum and urine are missing. Of note, an increase in autoantibodies may be caused by multiple factors including environmental triggers, such as a viral illness or a prolonged exposure to certain toxic chemicals.

In vivo, GTX exposure worsened the phenotype of an experimental autoimmune encephalomyelitis (EAE) model by triggering neuroinflammation and demyelination [58] supporting the hypothesis of an association between mycotoxin exposure and MS aggravation (Table 1).

Next to neuronal autoimmunity effects additional observations regarding autoimmune abnormality have been seen regarding fungal and mycotoxin exposure: in a small case control study the elevated levels of antigenicity for antimitochondrial antibodies (AMA) in 6 patients were all associated with mold and moisture exposure [59]. Mold-derived mycotoxins might induce mitochondria damage [60] and trigger autoimmunity via AMA, which are e.g., detected in more than 90% of patients with primary biliary cirrhosis [59].

For rheumatoid arthritis (RA), another autoimmune disease, only limited data are available analyzing the effect of mold exposure in humans [61]. A case study published in the 80s with only a limited number of patients described a tendency of stronger sensitization to *Aspergillus* spp. antigens in RA patients compared to controls. There are currently no further human data available for a decent assessment of a potential relationship between RA and mold exposure [62]. However, in an experimental RA model OTA and DON have been examined, showing that both mycotoxins have the potential to increase the susceptibility and severity of RA [63]. The exposed mice showed an enhanced clinical score for each paw with histopathology showing infiltrated leucocytes, synovial hyperplasia, pannus formation, cartilage destruction and bone erosion [63].

### 3.3. Mycotoxin Exposure Is Associated with an Onset of Inflammatory Bowel Disease

The incidence of inflammatory bowel diseases (IBD) is increasing in Western and developing countries. IBD are multifactorial disorders involving complex interactions between genetic, immune, and environmental factors such as exposure to food contaminants. Previously, IBD has been classified as autoimmune diseases, but new research has shown, that inflammation is rather caused due to an immune barrier defect [64].

Inflammagens inducing an aberrant immune response have long been hypothesized as trigger for the development of chronic inflammatory diseases [65,66]. Brewer et al., 2013 presented a series of human case reports providing evidence that molds and released mycotoxins act as inflammagens possibly residing in patients with chronic illness and contribute to its chronic progression [66]. This hypothesis is partly supported by a recently published human case report, presenting a 25-year-old male patient with a refractory ulcerative colitis (CU), chronic fatigue syndrome, and an HLA-DR/DQ genetic background, who was tested positive for mycotoxin of trichothecene (Table 1) [65]. In this case report de-challenge of mold exposure resulted at least in recovery of acute pancreatitis symptoms although colitis itself needed further medical treatment [65]. The strength of this case report is the test for mycotoxin and the follow-up after de-challenge. This approach is recommended for future cohort studies. Nevertheless, patients with comorbidities next to IBD should be excluded to avoid disturbance by concomitant disease when investigating mycotoxin effects in IBD patients.

Stronger evidence is provided by a well-designed in vivo experiment showing that mycotoxins DON [67] and zearaleone (ZEA) [68] have the potential to induce the onset of colitis showing morphological changes and increased colonic inflammation. Next to their potential to induce or trigger IBD in vivo, mycotoxins also worsened colitis symptoms after DON exposure in a colitis rat animal model (Table 1) [67].

Taken together, there are currently only few data available indicating that mycotoxins might be a relevant risk for the onset or exacerbation of IBD in humans. Therefore, epidemiological studies are necessary to further investigate the role of mycotoxins as a relevant risk factor for IBD and other immune-related intestinal disorders.

### 3.4. Mycotoxin Exposure and Human Immunodeficiency Virus (HIV) Infection

So far, two studies have been published supporting the hypothesis that mycotoxin exposure might have the potential to worsen human immunodeficiency virus (HIV) infection. It has been demonstrated that a correlation exists between an increased HIV burden and elevated aflatoxin B1 albumin adducts (AF-ALB) in the serum of HIV-positive individuals (Table 1) [69,70]. Similarly, a positive correlation between OTA plasma levels, HIV target cell activation, and plasma levels of the pro-inflammatory chemokine chemokine (C-X-C motif) ligand 10 (CXCL10) has been shown in a cohort study with children exposed to HIV. These findings indicate that OTA exposure has the potential to increase immune activation and is especially harmful for HIV-exposed or HIV-infected individuals (Table 1) [45].

Since AF and OTA exposure, as well as HIV-infection, are known to cause immune modulation [47,71,72], a synergistic relationship may exist and is postulated [73]. Although, these studies indicate a possible correlation, there are no in vivo or in vitro studies for a decent clarification of underlying pathophysiological mechanisms. However, the positive correlation between increased mycotoxin levels and HIV load might not be of a causative nature, but rather reflect the high prevalence of HIV-infection and missing food controls in the living area of the observed population. Pointing towards a poor health and food supply system.

**Table 1:** Mold-induced exacerbation of underlying disease.

Mold	Mold Component	Species	Disease/ Model	Measured Surrogate Marker	Outcome	Source
Increased Asthma severity						
*A. fumigatus*	Spores	Human	Asthma	Total serum IgE ↑ Blood eosinophils (cells/µL) ↑ FEV1/FVC (%) ↓	Asthma severity increased	[50]
*Penicillium* spp.	Spores	Human	Asthma	FEV1/FVC (%) ↓	Asthma severity increased	[50]
n.a.	GTX Patulin	Mouse	Allergic asthma/ OVA-model	Airway inflammation ↑ OVA-IgE ↑ BAL eosinophils ↑	Asthma severity increased	[54]
Involved in autoimmune response						
Mold water damage	n.a.	Human	n.a.	IgG neuronal antibodies against microtubule-associated protein-2, myelin basic protein, tau, glial fibrillary acidic protein, tubulin, and S-100B	No clincial outcome	[57]
n.a.	GTX	Mouse	EAE	Neuroinflammation ↑ Demyelination ↑	Aggravation of autoimmune encephalomyelitis	[58]
*A. fumigatus*	*A. fumigatus* antigens (I and VIII)	Human	RA	IgG ↑ IgA ↑	Stronger sensitization than control subjects	[62]
n.a.	OTA DON	Mouse	RA DBA1 model	IgG1 ↑ IG2a ↑ Pro-inflammatory cytokines ↑	Clinical severity score ↑	[63]
Trigger of IBD						
*S. chartarum*	Trichothecene group	Human	CU/CSF HLA-DR/DQ susceptible	Mycotoxin test in urine-positive	Pancreatitis improved after withdrawn	[65]
n.a.	DON	Rat	CU DSS model	Morphological damage in colon ↑ Colonic inflammation ↑	Exacerbation of onset and symptoms of DSS-induced colitis	[67]
Worsening of HIV condition						
*Aspergillus* spp.	AFB1	Human	HIV-positive adults	Plasma aflatoxin B1 ↑ HIV-1 RNA ↑	Higher viral loads in HIV-positive humans with higher AF-ALB	[69,70]

## 4. Mode of Action

The immune system plays a central role in mold-host interaction. Several organ-specific barrier functions are impaired by mycotoxins [71,74,75]. Additionally, immune response against mold has been shown to be species- and mycotoxin-dependent [72]. Especially trichothecenes, AF, GTX, ZAE, citrinin, fumonisins, ergot alkaloids, OTA, and patulin have been studied in vivo and in vitro to identify cellular targets and molecular mechanisms of action [22]. Brown et al., 2021 [76] summarized in detail how various mycotoxins affect different immune cells under physiological conditions and concluded that the effects on immune cells exerted by mold toxins most commonly involve the induction of apoptosis and the suppression of specific immune cell function.

Herein, we summarize the immune response to mold and mycotoxins under different impaired immune system conditions. Therefore, we focus on in vivo and in vitro models to sum up and partly discuss the current knowledge of cellular and molecular modes of action.

### 4.1. Effects of Mold on the Immune System

Mold species and its components including mold spores and hyphal fragments have been recognized to be involved in inflammation and as allergens that can cause different diseases [31]. The immune system is triggered by several mycelium molecules of molds. β-1,3 glucans are naturally occurring in the cell walls of fungi and are recognized by host phagocytic cells, dendritic cells, neutrophils, and epithelial cells via C-type lectin receptors (dectin-1 and 2, Mincle and mannose receptors) [77]. Moreover, mold cell wall is composed of glycoproteins with allergenic properties [78]. In addition, mold allergens are also found in enzymes released in the external environment during the germination process [79,80]. The lung epithelium and the dendritic cells present in the lung can recognize these allergens which initiates the allergenic process. Findings show that C-type lectin receptors can activate innate immune cells, the first line of defense, and modulate the development of the adaptive immunity through the differentiation of T-helper 1 (Th1) and T-helper 17 (Th17) cells, two critical cell subsets to mount a protective immune response against pathogenic fungi [81,82,83]. These responses have been widely investigated with the pathogenic yeast *C. albicans* and the mold *A. fumigatus* but the relative contribution of C-type lectin receptors in the development of immune responses against nonpathogenic molds as for instance *Penicillium* spp., *Cladosporium* spp. or *A. alternata* is lacking and the mechanisms involved are not formally identified yet.

Moreover, the engagement of C-type lectin receptors triggers the activation of intracellular signaling pathways leading to the activation of inflammasomes and promotes inflammatory responses critically required to control fungal infections, including mold [84]. For A. fumigatus it has been shown that C-type lectin receptor dectin-1 at the surface of dendritic cells induces respiratory burst and the production of inflammatory mediators, including tumor necrosis factor-α (TNF-α), various interleukins (IL-1β, IL-6, IL-23), chemokines ligands (CCL-2 and CCL-3), and chemokine (C-X-C motif) ligand 1 (CXCL-1) [85]. When knocking out dectin-1 in mouse models mortality rate increases due to poor neutrophil recruitment and an impaired fungal killing of *A. fumigatus* [86].

Next to the discussed mold components, mycotoxins as secondary metabolites of molds have been shown to have multiple mode of actions including impairment of barrier functions and exacerbation of inflammation as presented in the following sections.

### 4.2. Mycotoxins Are Systemically Bioavailable

The route of mold and mycotoxin exposure might be relevant for the discussion of local effects and adverse reactions. However, it is important to mention that mycotoxins are systemically bioavailable irrespective of the route of exposure. Mycotoxin exposure via the airways results in systemic bioavailability in animal models [87]; multiple organs are therefore potentially exposed [88,89]. Even oral mycotoxin intake resulted in chronic inflammation of the lungs [90,91].

### 4.3. Mycotoxins Compromise Barrier Functions

Epithelia and the mucosal barrier build the initial defense shield against molds, irrespective of exposure route. Intestinal epithelial cell death or alterations of the tight junction can be causative for transcellular and paracellular transport. Intestinal barrier integrity is impaired by e.g., *A. flavus* toxins AFB1 and AFM1 [92], which has been shown for differentiated gastrointestinal epithelial cells [75,93].

When mycotoxins are inhaled, they locally affect the respiratory epithelium and increase the invasive capacity of molds. For GTX in vivo experiments in mice demonstrated that the invasive capacity of *A. fumigatus* was increased and even correlated with increased mortality [94,95].

This is in accordance with in vitro experiments of human alveolar epithelial cells. In human bronchial epithelial cells GTX promoted cytoskeletal remodeling, which facilitates the internalization of *A. fumigatus* [94,95]. Other in vitro experiments were designed to test effects of acute exposure to AF on airway cell physiology, showing that AF reduced the respiratory mucosal ciliary function [96]. Moreover, sterigmatocystin (ST) induced apoptosis in human pulmonary cells in vitro [97]. Even alterations in blood brain barrier (BBB) permeability upon exposure to GTX have been described by using microvascular endothelial cells originated from human induced pluripotent stem cells (iPSCs) [98].

Taken together, these findings implicate mycotoxins facilitate the entrance of mold components like inflammagens and/or allergens by compromising of the barrier function resulting in an inappropriate immune response.

An impaired epithelial barrier function is discussed in various diseases like asthma, IBD and MS to be a risk factor for inflammatory changes. In asthma, airway epithelium is dysfunctional since tight junction formation is interrupted [74]. Dysfunction of epithelial barrier and its aggravation are thought to have a significant pathophysiological contribution in asthma pathophysiology [99].

Moreover, in IBD, the breakdown of intestinal epithelia barrier function is characteristic for the diseases [100]. Although clinical and experimental data implicate a causality between intestinal hyperpermeability and IBD, so far no direct causal relationship was confirmed [101].

Furthermore, in MS the BBB function is altered. A recently published review summarizes pathophysiological studies of MS patients, showing BBB abnormalities and a strong correlation between inflammation and degeneration in MS progression [71].

In summary, there is evidence that epithelial barrier dysfunction is an important risk factor for inflammatory changes in various chronic inflammatory diseases and future research should address the role of mycotoxins as potential trigger and/or booster in adequate in vivo diseases models.

### 4.4. Mycotoxins Can Influence the Gastrointestinal Microbiota

The gut microbiome regulates the intestinal homeostasis and is an important part of the mucosal immune system [102]. Inflammation processes have been shown to result in dysbiosis having the potential to induce intestinal diseases [71,102] and autoimmune disorders [103]. It has been demonstrated that mycotoxins including trichothecenes, ZAE, fumonisins, OTA, and AF can influence the gastrointestinal microbiota directly through antimicrobial activity and through secondary mechanisms involving the release of antimicrobial compounds from mycotoxin damaged host cells [104,105]. So far, it has been identified that the interaction between gut microbiota and mycotoxins plays a significant role in the development of mycotoxicosis [104]. As already discussed, DON is a risk factor for IBD onset [67]. Chronic treatment of rats with DON over 9 months with doses relevant for currently estimated human diet exposure resulted in a reshaped gut microbiota and dysbiosis [27]. Furthermore, DON exposure exacerbated the genotoxic effect of *E. coli* strains producing colibactin in the gastrointestinal tract [106]. Colibactin is a genotoxic secondary metabolite produced by a subset of phylogroup B2 *E. coli* strains and associated with DNA double-strand breaks in intestinal epithelial cells [106].

These findings might even be a first hint at a synergistic effect between DON exposure, gut microbiota, and increased risk of intestinal carcinogenesis.

In general, it is most likely that a mycotoxin-induced gut dysbiosis might be a trigger for the development of various diseases.

### 4.5. Mycotoxins Have the Potential to Induce and Exacerbate Inflammation

Mycotoxins can initiate or enhance inflammation [76,107], and several molecular modes of action are considered like overactivation of inflammasome-mediated responses or the induction of ROS [108,109]. Receptor-mediated inflammasome activation has been shown for ZEA [68], AFB1 [110], and trichothecene [111]. In addition, patulin induction of pyroptosis through autophagic inflammasomal pathway has been demonstrated in liver cells [112].

In autoimmune diseases, inflammatory processes e.g., neuroinflammation in MS are key mechanisms to pathophysiology. Environmental agents like food additives and pollutants or toxins, among which are mycotoxins, at prolonged low-dose-exposure conditions, might trigger the essential molecules associated with central nervous system (CNS)-immune system interactions.

**Figure 1:**
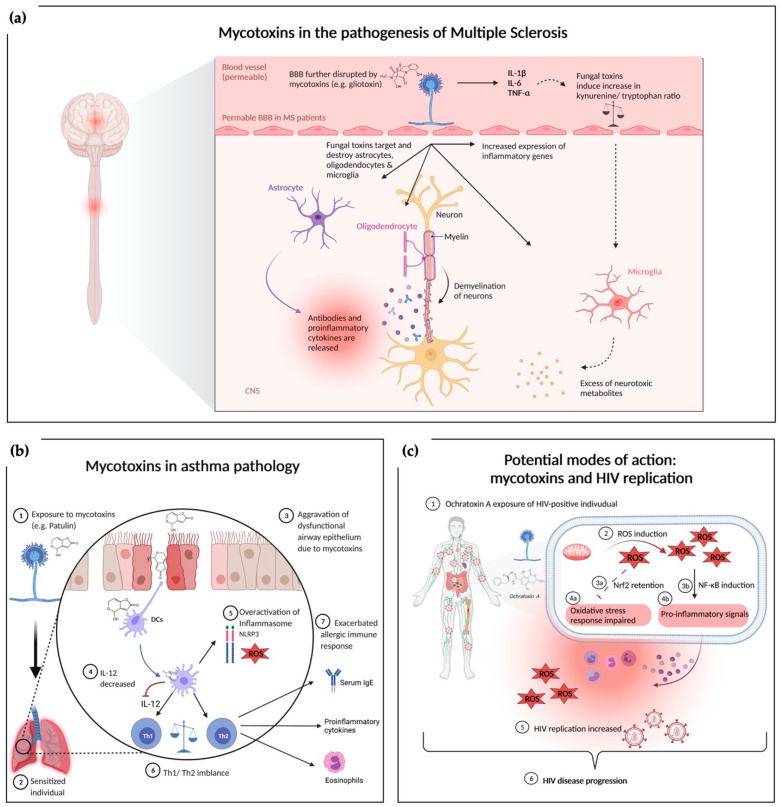
Overview of mechanisms underlying the exacerbating effects of mycotoxin exposure under a dysregulated immune system. (**a**) Mycotoxins in the pathogenesis of MS: exposure to GTX alters the blood brain barrier [98]. Mycotoxins affect neural tissue by damaging astrocytes, oligodendrocytes, and microglia. Loss of oligodendrocytes leads to further demyelination while targeted astrocytes release proinflammatory cytokines contributing to the neuroinflammatory environment. Another direct effect of mycotoxins is the induction of proinflammatory gene expression in the CNS. Indirect pathways via proinflammatory cytokines like IL-1β are hypothesized to interact with microglia through an augmented kynurenine/tryptophan ratio, which promotes the secretion of neurotoxic metabolites. (**b**) Mycotoxins in asthmatic conditions: exposure to mycotoxins worsens the respiratory epithelium barrier impairment. Dendritic cells take up mycotoxins leading to a decreased production of IL-12 and an increased ROS production as well as overactivation of the inflammasome. IL-12 reduction further emphasizes the Th1/Th2 imbalance contributing to the increased airway inflammation in asthmatic mouse model (**c**) Potential modes of action between mycotoxin exposure and HIV replication: exposure to mycotoxins modulates immune response via induction of ROS. ROS inhibits oxidative defense machinery via Nrf2 retention and induces proinflammatory response via NF-κB induction. Both, mycotoxin related oxidative stress and proinflammatory signals could potentially contribute to an increased HIV burden and disease progression. Figure 1a adapted from “Allergic airway sensitization”, by BioRender.com (2021). Retrieved from https://app.biorender.com/biorender-templates; Figure 1b adapted from “Pathogenesis of Multiple Sclerosis”, by BioRender.com (2021). Retrieved from https://app.biorender.com/biorender-templates.

The inflammatory aspect links these autoimmune diseases and nervous system disorders [113,114,115]. Traditionally, neuroinflammatory disorders like MS are characterized by leukocytes invading the CNS parenchyma and deliver cytokines to the tissue, inducing an inflammatory cascade. Interleukin-1 β (IL-1β) and IL-6 are central to the inflammatory process and can be sensed by astrocytes, microglia and monocyte-derived cells [116]. During the cytokine activation, tryptophan metabolism is also interrupted in favor of decreased serotonin availability and increased kynurenine, ROS, nitric oxide (NO), and glutamate release (an excitatory neuromodulator) [117,118]. The increased inflammatory mediator profile and kynurenine to tryptophan ratio suggest that inflammatory mediators might be responsible for a high risk of psychological disturbances [119,120]. Figure 1a summarizes the current knowledge of the complex interaction between mycotoxins and MS pathophysiology [121,122]. GTX has been shown to effect and damage microglial, astrocytes, and oligodendrocytes [123]. Direct interactions of fungi including molds with the CNS or indirect toxin release from a non-neurological site can be causative. Dose-dependent GTX increased neuroinflammation accompanied by aggravation of demyelination has been shown in a MS mouse model [58]. CNS analyses revealed that GTX locally increased the relative expression of inflammatory genes and cytokine production, thereby exacerbating neuroinflammation [58]. Different neurological diseases might be influenced by mycotoxins via similar mechanisms [58]. Immune alterations can induce many proinflammatory cytokines by peripheral blood mononuclear cell (PBMCs), endothelial cells, glial cells, and neurons in the Toll-like receptor 4 (TLR4)/nuclear factor kappa-light-chain-enhancer (NF-ĸB), and mitogen-activated protein kinases (MAPKs) dependent manner [123,124,125]. Such unbalanced metabolic and physiological status mediated by pathogen-associated molecular patterns (PAMPs), damage-associated molecular pattern (DAMPs), cytokines (TNFα, IL-6, IL-17, and IL-1β), immune cells upregulate TLRs, and cytokine receptors (G protein-coupled receptors and tropomyosin receptor kinase-A) influence neural circuits. Hence, a continuous immunological sensory flow involves the fundamental aspects of neuronal integrity, secondary messengers, calcium (Ca^2+^), cyclic adenosine monophosphate, and intracellular kinases [113,126].

Chronic inflammation is characterized by infiltration of affected organs by immune cells like neutrophils, eosinophils, macrophages and dendritic cells [127]. Cytokines, ROS and other proinflammatory mediators are secreted by these immune cells and released into blood circulation [127], potentially activating excessive inflammation [128], asthma [34], and IBD [129]. ZAE has been shown to induce intestinal inflammation in vivo under physiological conditions [68]. First evidence points out, that ROS-mediated nucleotide-binding oligomerization domain, leucine rich repeat and pyrin domain containing 3 (NLRP3) inflammasome activation might be the possible underlying mechanism [68]. In an animal model of IBD it has been shown that oral exposure to DON, induces intestinal breakdown and inflammatory response leading to CU [67]. DON increased morphological damage, proinflammatory markers (myeloperoxidase, CXCL-1) and IL-1β), and immune cell responses. In lamina propria of the rat CU models DON increased adaptive and innate immune responses after anti-CD3/28 or LPS stimulation, respectively. In the spleen, DON increased IFN-γ secretion and reduced Treg populations. Interestingly, De-epoxy-DON (DOM-1) a detoxified form of DON did not have any consequences on colitis [67].

### 4.6. Mycotoxins Interfere with T-Cell Differentiation

In an in vivo study, it has been clearly demonstrated that respiratory exposure to GTX and intestinal exposure to patulin exacerbated the asthma-like phenotype in the studied asthma-model (Figure 1b) [54]. The mechanisms of action for underlying exacerbating effects might be caused by the potential of GTX and patulin to interfere with Th1/Th2 balance [130,131]. Indeed, in an asthma model increased Th2 cytokine levels and a reduced production of the Th1 cytokine IFN-gamma was detected [54], which might possibly serve as an explanation for the exacerbating effect. Th1/Th2 imbalance is most likely connected to a reduction of IL-12 production by dendritic cells [54], without affecting cell viability [130,131]. Inhibition of IL-12 caused by mycotoxins might therefore be a key element leading to an impaired Th1 cell differentiation, promoting the development of Th2-driven asthma and other allergic disease. It has been shown that IL-12 production by dendritic cells of allergic patients is reduced compared to healthy subjects [132,133].

The role of chronic inflammation in the pathogenesis of inflammatory arthritis, for example, RA and spondylarthritis, is well established in that immune cytokines attributed to genetic and environmental factors drive systemic inflammation [134,135]. OTA and DON have been shown to exacerbate the severity of RA in an experimental mouse model [63]. This effect results through an enhanced stimulation of macrophages leading to an increased release of the pro-inflammatory cytokines IL-1β, IL-6 and TNF-α followed by a promotion of Th1/Th17 cell differentiation [63]. In addition, the production of IL-1β and IL-6 in inflamed joints and of IFN-γ and IL-17 in splenocytes were elevated [63].

### 4.7. Mycotoxins and Their Potential Exacerbating Effect Regarding HIV Infection and Diseases Progression

In the past years, several human studies (Table 1) supported the idea of a potential exacerbating effect of HIV progression due to mycotoxin exposure.

Since both, mycotoxins and HIV cause immunosuppression, chronic exposure to mycotoxins in HIV-positive people could lead to higher levels of viral burden and disease progression. Mycotoxins like OTA [136], AF [41], and trichothecenes [137] have the potential to induce immunosuppression. Multiple cellular targets have been described for these toxins. Among others inhibition of protein synthesis by ribosome targeting, and disruption of mitochondrial function has been shown [138,139,140,141]. In vitro OTA-induced immunosuppression was recently demonstrated [136]. Long-term instead of short-term exposure induced immunosuppression, which involved inhibition of autophagy through upregulating p-Akt1 [136]. In an in vivo mouse model AFM1 exposure resulted in a lower mass of spleen, indicating an overall lower number of T and B cell subpopulations [41]. It was hypothesized that this effect might be related to ROS and formation of DNA adducts (AFM1-N7-guanin) [41]. A recent review from Wong et al., 2016 [137] mentioned, that trichothecenes can cause immunosuppression in lymphocytes [142].

Past studies showed significantly lower percentages of CD4^+^ T cells [69,143] and B-cells [143] in HIV-positive patients with high AF-ALB compared to HIV-positive patients with low AF-ALB levels, indicating a synergistic immunosuppressive effect of mycotoxins and HIV infection.

However, recent human studies showing increased mycotoxin levels and elevated HIV burden, while CD4^+^ T cell numbers were still in normal range [70]. In another study, levels of HIV target cells and CD4^+^ T cell activation was increased, which is important in regard to HIV replication [45]. Moreover, the inflammatory marker CXCL10, which is associated with Th1 T cell mobilization and activation was significantly increased [45].

Regarding these findings, a modulation of the immune response by mycotoxins resulting in increased HIV burden and HIV disease progression (Figure 1c) is currently hypothesized. Modulation of the immune response by OTA is believed to be mediated in part by the induction of reactive oxygen species [45]. OTA can induce one or more critical redox signaling molecules including NF-κB and NF-E2-related factor 2 (Nrf2) via ROS [144]. It has been shown that OTA-induced ROS and oxidative stress is accompanied by an imbalance in the cellular oxidative defense machinery via retention of Nrf2 [144]. Simultaneously, NF-κB is induced, indicating its role in a proinflammatory response [144]. Interestingly, low dose OTA exposure in pigs was associated with increased replication of porcine circovirus type 2 (PCV2), possibly mediated by intracellular redox status accompanied by intact cell viability of treated cells [145]. These observations are important, when speculating about a possible underlying mechanism of an exacerbating effect of OTA exposure in HIV-positive individuals. ROS production and oxidative stress caused by mycotoxins are accompanied by proinflammatory response and immune stimulation, which potentially promotes HIV replication [146]. There are several studies discussed in a recent review, which together demonstrate that especially chronic oxidative stress has detrimental consequences to HIV related immune response, resulting in an impaired capability to adequately respond to viral replication [146].

In addition, stimulation of proinflammatory pathways due to impaired oxidative stress response might be crucial [144,145]. Proinflammatory cytokines have multiple modes of action including: regulation of HIV replication, influencing HIV lifecycle, the establishment of latent HIV reservoirs, and chronic inflammation can contribute to apoptosis of CD4^+^ and CD8^+^ T cells resulting in immune suppression and disease progression [147].

According to available data, it can be hypothesized that mycotoxin exposure in HIV-positive individuals exacerbate HIV burden in acute phase—at least in part via ROS, enhancing oxidative stress and inflammatory response, while chronification of inflammation during the chronic phase might then enhance immunosuppression and disease progression.

## 5. Conclusions and Future Perspectives

Mold and mycotoxin exposure results mainly from contaminated food and inhalation of spores. Mold spores range in their size from 3 to 40 µm allowing them to flow in the air and being inhaled by animals and humans. Spores serve as vectors for mycotoxins and are pathogenic due to allergenic and infectious properties. Irrespective of exposure route, mycotoxins are systematically bioavailable affecting different system organ classes. In immunocompetent individuals, direct allergenic, and immune modulatory effects of mycotoxins are well described and accepted. In contrast, from a clinical point of view the concept of toxic mold syndrome that might be directly caused by mold or mycotoxins is discussed controversially. Moreover, according to critical opinions there is no sufficient evidence that mold/mycotoxin exposure might directly induce autoimmune diseases.

The situation appears different considering the role of mold and in particular of mycotoxins as risk factors in the onset and severity of various diseases in individuals with an already impaired immune system. Exacerbation of asthma has already been shown in well-designed human cohort studies, meta-analyses, and animal models. Although there seems to be an association between mold/mycotoxin exposure and exacerbation of other dysregulated immune conditions like inflammatory bowel diseases, autoimmune diseases or disease progression in HIV-positive patients, no causality could be demonstrated to date.

Epidemiological studies are important for the estimation of risk factors regarding diseases. Future human cohort studies regarding immune system disorders including chronic inflammatory disease, autoimmune diseases, and HIV disease should consider environmental chemicals like mycotoxins to a greater extend to evaluate the actual relevance for human disease progression.

However, to determine a causal relationship randomized controlled double blind interventional human studies are needed, which are unethical and therefore justifiably not allowed. Therefore, well-conducted in vivo and in vitro research studies with plausible explanations of pathological mechanism should be performed. Additionally, the invasion of mold under such conditions should be addressed as well. Although emerging evidence outlined in the current review clearly indicates that mycotoxins have an exacerbating effect on pre-existing immunological disorders, the key elements have yet to be determined. To do so, we encourage future research to consider the following points:

(1) Study objectives should include investigation of the adjuvant activity of mycotoxins of the addressed immune system disorders. For better understanding of the underlying pathological mechanism, future research should investigate the key modes of action including the alteration of barrier functions, exacerbation of inflammation and T-cell differentiation. Moreover, the measurement of relevant disease parameters like the influence on gastrointestinal microbiota in IBD, or axonal demyelination in MS is strongly recommended.
(2) Using appropriate disease models for a valuable translation to human exposure and clinical research is suggested. Therefore, it is necessary to determine state of the art disease models and use disease-specific biomarkers as readout. Real-world human mycotoxin exposure scenarios should be mimicked in model organisms to be able to make estimations about critical threshold concentration for specific vulnerable populations.
(3) Investigations to develop prevention or treatment strategies to face the exacerbation of pre-existing immune system disorders due to mycotoxin contamination should be addressed as well. It should be questioned if the harmful effects are reversible after elimination of the mycotoxin or by interfering with the mycotoxin modes of action. E.g., the development of sensitive and specific detection assays for multiplex mycotoxin detection could be established for comprehensive diagnostics.
(4) Next to mycotoxin exposure, also invasion mechanisms of mold might be of interest depending on the disease model. Under this scope, antifungal treatment options should be investigated as well.

These different research approaches are needed to complement the current clinical knowledge and link the dots until well-studied interventions are available to properly assess the combination of concern—mold, mycotoxins and a dysregulated immune system.

## Data Availability

Not applicable.
